# In Vitro Antibacterial, Antioxidant, Anticholinesterase, and Antidiabetic Activities and Chemical Composition of *Salvia balansae*

**DOI:** 10.3390/molecules28237801

**Published:** 2023-11-27

**Authors:** Amırat Mokhtar, Tabak Souhila, Bouriah Nacéra, Benabdallah Amina, Mohammed I. Alghonaim, Mehmet Öztürk, Sulaiman A. Alsalamah, Mohamed Djamel Miara, Fehmi Boufahja, Hamdi Bendif

**Affiliations:** 1 Institute of Veterinary Sciences, University Ibn-Khaldoun of Tiaret, Tiaret 14000, Algeria; herpetowalid5@gmail.com; 2 Department of Nature and Life Sciences, Faculty of Life and Nature Sciences, University of Tiaret, Tiaret 14000, Algeria; biologi4000@hotmail.fr (T.S.); nacera.bouriah@univ-tiaret.dz (B.N.); miara14130@yahoo.fr (M.D.M.); 3 Department of Agronomy, Faculty of Life and Nature Sciences, University Chadli Bendjedid, El-Tarf 36000, Algeria; benabdallah-amina@univ-eltarf.dz; 4 Biology Department, College of Science, Imam Mohammad Ibn Saud Islamic University (IMSIU), Riyadh 11623, Saudi Arabia; mialghonaim@imamu.edu.sa (M.I.A.); saalsalamah@imamu.edu.sa (S.A.A.); 5 Department of Chemistry, Faculty of Sciences, Muğla Sıtkı Koçman University, Muğla 48121, Türkiye; mehmetozturk.dr@gmail.com; 6 Laboratory of Ethnobotany and Natural Substances, ENS de Kouba, Algiers 16308, Algeria; hamdi.bendif@univ-msila.dz; 7 Faculty of Sciences, University of M’sila, P.O. Box 166, M’Sila 28000, Algeria

**Keywords:** antibacterial, antioxidant, anticholinesterase, antidiabetic, chemical composition, *Salvia balansae*

## Abstract

Context: *Salvia balansae* de Noé (*S. balansae*) (Lamiaceae) is known to be an important plant used in folk medicine as an herbal remedy in Algeria. Objective: The purpose of the present study was to demonstrate the phytochemical composition, antioxidant activities, enzyme inhibitory activities, and antimicrobial activities of *S. balansae* extracts. Materials and methods: A methanolic extract and a petroleum ether extract from the aerial parts of the plant were assessed for their chemical composition. HPLC-MS and HPLC-DAD assessed the content of phenols, GC-MS the fatty acid composition, and ICP-MS the mineral profiles of the plant. Additionally, we evaluated the bioactivities of *S. balansae* extracts by the DPPH, ABTS, and CUPRAC assays, including the antioxidant potential against AChE, BChE, α-amylase, and α-glucosidase for enzyme inhibition. The antibacterial and antifungal activities of the methanolic extract were determined by the disc diffusion test against several strains of bacteria and yeasts. Results: Our findings revealed that the aerial parts of *S. balansae* were rich in phytochemical components and contained large amounts of minerals. Quantitative analysis of phenolic compounds by HPLC-DAD revealed the presence of 12 compounds in three major classes, flavonoids, hydroxycinnamic acid, and phenolic acid derivatives, with 0.61, 0.45, and 0.29 mg/g of extract, respectively. Nine phenolic constituents were quantified by HPLC-MS analysis; catechin (72.5%) was the main compound, followed by myricetin (21.7%). The fatty acid composition of the *S. balansae* petroleum ether extract by GC-MS analysis was quantified. Seventeen compounds, including palmitic acid, were identified as the major fatty acids. The antioxidant activity of the *S. balansae* extracts was measured by three different methods: the methanol extract provided better results than the petroleum ether extract, and interesting values were noted for the DPPH, ABTS, and CUPRAC assays of 242.7 ± 7.44, 124.1 ± 9.70, and 222.9 ± 6.05 µg/mL, respectively. The enzyme inhibition activity of the plant could not be determined. The antimicrobial results of the methanolic extract obtained from the disc diffusion method, followed by measurements of MIC, MBC, and MFC against several bacteria and yeasts, indicated that *S. balansae* exhibited noticeable antimicrobial and antifungal activities. Conclusions: These results provided new data about the main phenolic compounds and biological activities of extracts of the aerial parts of *S. balansae*, which might be an alternative source for synthetic bioactive compounds.

## 1. Introduction

Plants have always been important for humans, particularly for their nutritional and healing properties [1]. In the last decades, the production and analysis of medicinal plants became the ultimate goal of researchers worldwide, who hoped to use them to avoid the need for chemical therapies and their side effects in humans [2]. In recent times, herbal remedies have become more and more popular and more frequently used because of their lack of unsafe effects. The production of chemical-free products from medicinal plants has gained more attention because of their quality and safety [3]. They were tested by our ancestors, and many of their virtues occupy an important place in traditional therapy. Medicinal plants remain a source of medical care in developing countries in the absence of a modern medical system [4]. It is known that more than 80% of the world’s population still uses medicinal plants for health purposes [5]. In addition, more than 25% of modern medicines contain one or more active ingredients of plant origin [6]. Indeed, it is hoped that the investigation of medicinal plants will provide permanent remedies for different diseases and that the discovery of new active ingredients and/or new biological activities of the chemical components synthesized by these plants will be valuable in treating ailments [7].

The Lamiaceae family is widely distributed over almost the entire earth, with a denser presence in the Mediterranean and subtropical regions. The genus *Salvia*, its name derived from the Latin word *Salveo*, meaning “to save”, probably because of the curative virtues of some of its species, was known throughout antiquity. *Salvia*, commonly known as sage, appears to be one of the oldest plants used in traditional medicine over time. Besides their medicinal properties, sage plants are also known for their cosmetic value, gourmet flavors, apicultural quality, ornamental attraction, and ecological importance [8]. Algeria is an integral part of the biodiversity hotspot in the Mediterranean region [9]; it is recognized as an important center of plant species diversity and endemism [10]. There are 19 species of *Salvia* in Algeria, of which the majority are concentrated in the northern part of the country [11]. They grow in grasslands, among trees and shrubs of uncultivated fields, on hillsides, in rocky places, on wadi banks, in scrublands and forest clearings, and in clay or sandy soils from near sea level up to 2000 m in altitude. They can be annual to biannual herbs, herbaceous perennials, or small woody shrubs. They have been categorized into five sections: section Eusphace (three species), section Horminum (one species), section Aethiopis (four species), section Plethiosphace (nine species), and section Notiosphace (two species). Among the five North African endemics present in Algeria (namely, *Salvia jaminiana* de Noé, *Salvia pseudojaminiana* Chevall., *Salvia algeriensis* Desf., *Salvia balansae* Noë ex Coss, and *Salvia chudaei* Batt. & Trab.), only *S. jaminiana* and *S. balansae* are restricted to Algeria [12], and both are listed as protected species under Algerian Republic Law [13]. Only *S. balansae* is mentioned as “rare” in the IUCN Red List of Threatened Species [14]. The genus *Salvia* remains the most diverse in the Lamiaceae family, whose species are known worldwide due to their medicinal and culinary uses [15]. *S. balansae* Noë ex Coss. is a rare endemic medicinal plant species limited to northwest Algeria. Originally from the region of Mostaganem, this species is noted in the floras as endemic to Algeria but in somewhat separate areas [12]. It is found in the lower Cheliff Valley near Mostaganem on the one hand and the Abdi Valley in the Aurès on the other hand, which is more than 500 km east and 1000 m higher than the Chelf Valley. The only commonality between these two sites is the semi-arid bioclimate, although one is in a maritime region with mild winters and the other is a region with cool winters [16]. *S. balansae* is an aromatic plant belonging to the Lamiaceae family; it is a shrub that is 30 to 70 cm, with linear leaves split into two deep lips and a corolla of more than 2 cm. *S. balansae* Noë ex Coss. is strictly endemic to Algeria, inhabiting scrubland located on the edges of valleys. It is an endemic and rare plant, known only in two stations in the northwest (Dahra) and east in the Aures [12]. According to Codd [17], most native *Salvia* species are known locally for specific traditional uses related to their countries of origin. However, several species, particularly *Salvia officinalis*, remain more or less known worldwide because they are cultivated all over the world. It is indeed historically known that certain populations, such as European descendants in South Africa, used an endemic species of sage in the same way and for the same purposes as *S. officinalis* [18]. These species’ most common medicinal uses are to treat colds, coughs, and bronchial infections. *S. balansae* is not known for particular ethnobotanical reasons but appears to be popular locally for uses similar to common sages. Phytochemically, *Salvia* species are mainly composed of phenolic acids, flavonoids, saccharides, diterpenoids, and triterpenoids [19,20]. Flavonoids, triterpenoids, and monoterpenes are mostly concentrated in the aerial parts of the plants, especially in the flowers and leaves, while diterpenoids and phenolic acids are found in the roots [21]. Also, *Salvia* species have many biological activities as antioxidants, antimicrobials, and anticancer substances because of their bioactive constituents [18,22,23]. These activities justify many of their traditional uses, particularly against cardiovascular, immune, hepatic, and renal system diseases [24,25]. However, unlike the cosmopolitan sage species or those with an average global distribution, the endemic taxa remain poorly known, particularly at the phytochemical and pharmacological levels. This is the case for *S. balansae*, for which we have been able to find only a few minor works describing some of these chemical and pharmacological properties. Indeed, to the best of our knowledge, only two studies of this species exist. One is by Mekki et al. [26] and deals with the therapeutic effects of the species on metabolic disorders and testicular dysfunction mediated by a high-fat diet in Wistar rats. The second was published by Mahdjoub et al. [27], and in it, the phenolic compounds of *S. balansae* leaves were extracted, separated, and identified by high-performance liquid chromatography with diode array detection (HPLC-DAD) with antioxidant activity evaluation. These studies constitute the first contributions to the knowledge about the chemical composition of this species, as well as its biological activities. Therefore, the objective of the present study was to investigate the bioactive compounds of this rare and endemic species in terms of phytochemical screening, mineral contents, HPLC-DAD analysis, HPLC–mass spectrometry (HPLC-MS) analysis, and fatty acid analysis by gas chromatography–mass spectrometry (GC–MS), plus evaluation of the antioxidant, enzyme inhibition, and antimicrobial activities of this species, in order to explore its potential applications for the food and pharmaceutical industries.

## 2. Results and Discussion

### 2.1. Phytochemical Screening

Phytochemical screening results for the methanolic extract of the aerial parts of *S*. *balansae* are shown in Table 1.

Highlighting different classes of botanical constituents can give us a better understanding of plant phytochemistry. According to the results of the phytochemical screening of *S. balansae*, tannins, phenolic compounds, flavonoids, quinones, anthraquinones, saponins, and reducing compounds were detected in varying degrees in the methanolic extract. Small amounts of terpenoids were also detected in the extract. Phytochemical screening results showed that the methanolic extract of the aerial parts of *S. balansae* was rich in phytochemical components.

### 2.2. Mineral Content

Many studies focused on the phytochemical screening of plants, but to the best of our knowledge, there are no published reports on the mineral quantifications of *S. balansae*. It is worth mentioning that the elemental mineral contents of the aerial parts are reported in our work for the first time. The mineral composition was determined using inductively coupled plasma-optical emission spectrometry (ICP-OES). According to the results, *S. balansae* contains high amounts of minerals (Table 2).

The results give the nutrient values in percentages of dried weight for the macrominerals phosphorus, potassium, calcium, and magnesium and in mg L^−1^ (parts per million) of dried weight for the microminerals iron, copper, manganese, zinc, and boron. Our findings revealed that the aerial parts contained large amounts of minerals, particularly iron, zinc, manganese, boron, copper, potassium, and calcium. Iron was the most abundant microelement present, at a concentration of 1160.36 ± 23.20 mg L^−1^, followed by manganese, zinc, boron, and copper. Potassium was the most abundant macroelement present, at a concentration of 2.38 ± 0.07%, followed by calcium, at a concentration of 1.52 ± 0.15%. The minerals mentioned above have a significant role in the functioning of the human body, and this makes *S. balansae* a good candidate for nutritional supplement products. More precisely, iron plays a crucial role in preventing anemia by synthesizing hemoglobin and myoglobin. It participates in many metabolic processes, including respiration and DNA synthesis; it is also responsible for enzyme formation, especially iron-containing enzymes [28]. Calcium is an essential element for building bones and teeth and ensuring the proper functioning of muscles and nerves [29]. Potassium has a role in protection against hypertension and improvement of bone health; is an essential dietary nutrient, constituting about 70% of the positive ions in cells; and is fundamental for regulating the acid–base and water balance of cells. It is crucial for heart and smooth muscle contraction, making it important for normal digestive and muscular function, while phosphorus is essential for skeletal mineralization [30]. Magnesium is involved in glucose homeostasis and has a significant impact on diabetes control [31]. It is a cofactor for many metabolic reactions and plays a vital role in bone mineralization and muscle relaxation [32]. It is worth mentioning that manganese, zinc, and copper are involved in tissue, cellular, and subcellular functions, including immune regulation through humoral and cellular mechanisms, nerve conduction, muscle contractions, regulation of membrane potential, mitochondrial activity, and enzymatic reactions [33], and they also play a key role in maintaining antioxidant defenses [34,35]. All of the minerals and trace elements measured are micronutrients that are essential for normal body function as they are beneficial for physiological functions [36]. These elements are involved in many biochemical reactions; they are present as stabilizing components of enzymes and proteins and function as cofactors for many enzymes [33]. Our findings agree with many studies previously mentioned that other *Salvia* species are rich in mineral compounds [37,38].

### 2.3. Quantitative Analysis of Phenolic Compounds by HPLC–DAD

**Figure 1:**
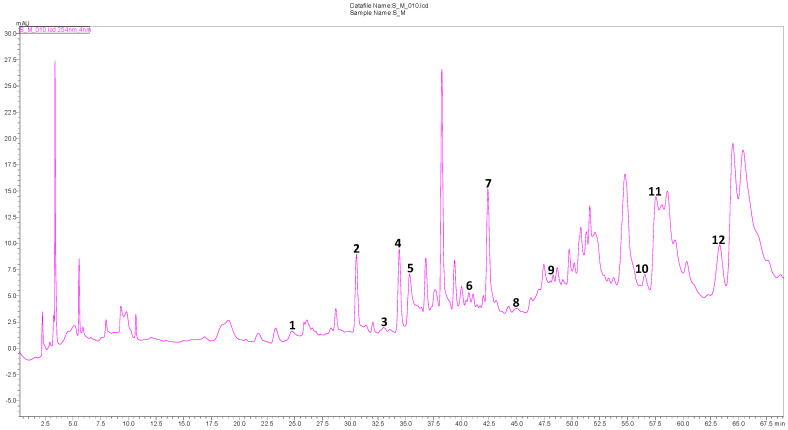
HPLC–DAD chromatogram of *S. balansae* methanolic extract at 254 nm (Inertsil ODS–3 olumn (4 μm, 4 mm × 150 mm). Mobile phase 0.1% acetic acid–methanol (gradient elution). Flow rate 1 mL/min. Diode array detection 254 nm.). **1**: Protocatechuic acid; **2**: *p*–Hydroxybenzoic acid; **3**: 6,7–Dihydroxycoumarin; **4**: Vanillic acid; **5**: Caffeic acid; **6**: *p*–Coumaric acid; **7**: Ferulic acid; **8**: Coumarin; **9**: *trans*–2–Hydroxycinnamic acid; **10**: *trans*–Cinnamic acid; **11**: Luteolin; **12**: Kaempferol.

Phenolic compounds constitute one of the key classes of secondary metabolites. They exhibit a huge variety of structures and are responsible for the major organoleptic characteristics of plant-derived foods and beverages, mainly color and taste. They also enhance the nutritional qualities of fruits and vegetables [39]. The HPLC-DAD identification of these compounds in our species led to the valuation of 29 diverse compounds and is presented in Table 3; the HPLC-DAD chromatogram of the methanolic extract of *S. balansae* is shown in Figure 1.

The compounds were identified by comparing their spectroscopic characteristics and retention times (RTs) with reference compounds. Twelve compounds were detected in low amounts ranging from 0.05 to 0.48 mg/g of extract (Table 3 and Figure 1). The phenolic composition was in three major classes: flavonoids, hydroxycinnamic acid, and phenolic acid derivatives, with 0.61, 0.45, and 0.29 mg/g of extract, respectively. The main phenolic compounds were luteolin (0.48 mg/g of extract), ferulic acid (0.45 mg/g of extract), vanillic acid (0.18 mg/g of extract), and kaempferol (0.13 mg/g of extract). Other compounds, such as 6,7-dihydroxycoumarin caffeic acid, *p*-coumaric acid, coumarin, *trans*-2-hydroxycinnamic acid, and *trans*-cinnamic acid, were detected in trace amounts (Table 3 and Figure 1). To the best of our knowledge, only the study of Mahdjoub [27] investigated the qualitative–quantitative analysis of phenolic compounds of an extract of *S. balansae* leaves. The HPLC-DAD analysis showed that there were 12 detectable phenolic compounds, and the total identified compound amounts ranged from 0.26 to 71.11 mg/g DW, representing two major classes: phenolic acids and flavonoids. The substances benzoic acid, quercetin, myricetin, hydroxyflavone, and ascorbic acid have been exclusively identified for the first time in *S. balansae* leaf extracts and have rarely been determined in other *Salvia* L. species. Other research has identified these compounds in several *Salvia* species in very low amounts [40,41,42,43].

### 2.4. HPLC-MS Analysis

**Figure 2:**
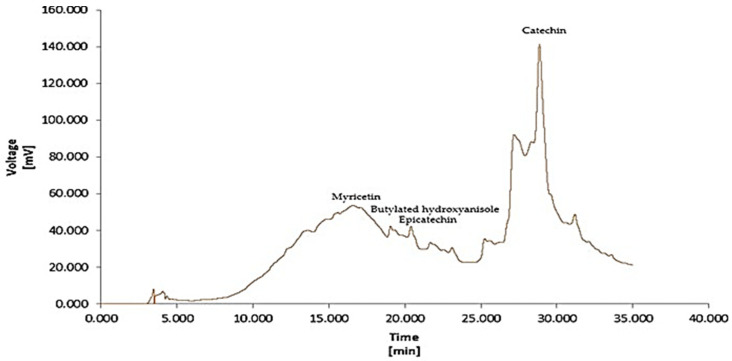
HPLC-MS chromatogram of the phenolic composition of the methanolic extracts of *S. balansae* represented the main compound.

Methanolic extracts of the aerial parts of *S. balansae* were subjected to HPLC-MS analysis, and nine phenolic constituents were quantified (Table 4 and Figure 2).

Catechin (72.5%, RT = 28.863) was the main compound, followed by myricetin (21.7%, RT = 16.590), while epicatechin (1.3%, RT = 20.403) and butylated hydroxyanisole (1.1%, RT = 19.060) were present in lower amounts. This was based on the only study recently published, conducted on the phenolic composition of the endemic species *S. balansae*. HPLC-DAD revealed the abundance of two main classes, phenolic acids and flavonoids, with high amounts of tannic acid, benzoic acid, gallic acid, and ascorbic acid. The flavonoids myricetin and catechin derivatives of the flavonoids myricetin and catechin were also identified as minor compounds [27].

### 2.5. GC-MS of Fatty Acid Composition

**Figure 3:**
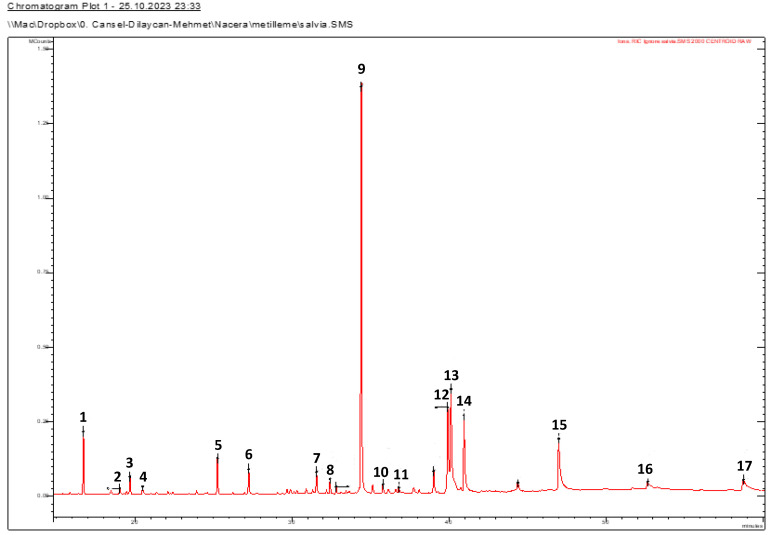
GC-MS chromatogram of the fatty acid composition of *S. balansae* petroleum ether extract. **1**: Dimethyl phthalate; **2**: Buthylatedhydroxytoluen; **3**: Dihydroactinidiolide; **4**: Nonanedioic acid (Azaleic acid); **5**: Isobutyl methyl phthalate; **6**: Myristic acid (C14:0); **7**: Phytone; **8**: Diisobutyl phthalate; **9**: Palmitic acid (C16:0); **10**: Isophytol; **11**: 15-Methyl-hexadecanoic acid; **12**: Lineloic acid (C18:2); **13**: Oleic acid (C18:1); **14**: Stearic acid (C18:0); **15**: Eicosanoic acid (C20:0); **16**: Docosanoic acid (C22:0); **17**: Hexacosane.

The fatty acid composition of the petroleum ether extract of *S. balansae* is shown in Table 5 and Figure 3. Seventeen compounds were quantified, and the degrees of unsaturation and saturation were 38.32% and 57.08%, respectively. Palmitic acid (C16:0) was identified as a major fatty acid; the other compounds were low in quantity. A polyunsaturated fatty acid concentration of *S. balansae* could lower the level of blood cholesterol and increase the nutritional value. Plants with a high concentration of unsaturated fatty acids are suggested for people with high cholesterol.

### 2.6. Biological Activities

#### 2.6.1. Antimicrobial Activity

**Figure 4:**
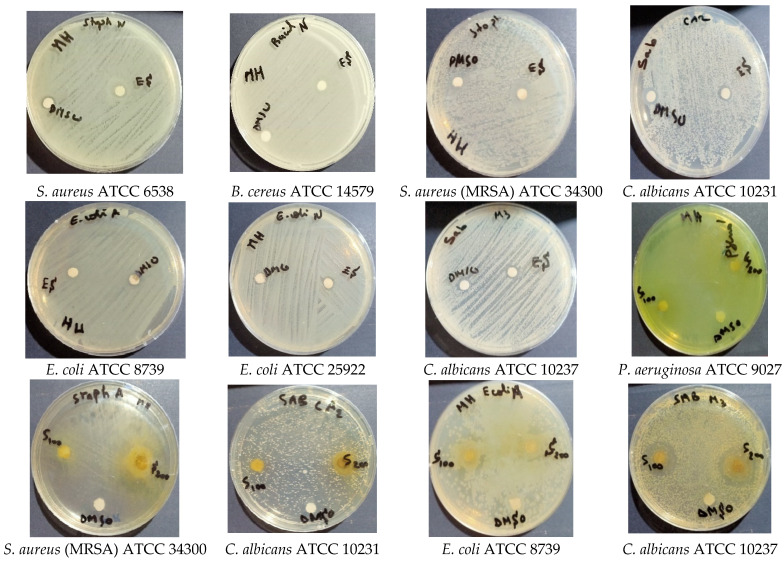
Antibacterial activity of *S. balansae* methanolic extract against the tested strains, diameter of inhibition zone (mm) for the concentration range of 50 mg/mL, 100 mg/mL, and 200 mg/mL.

**Figure 5:**
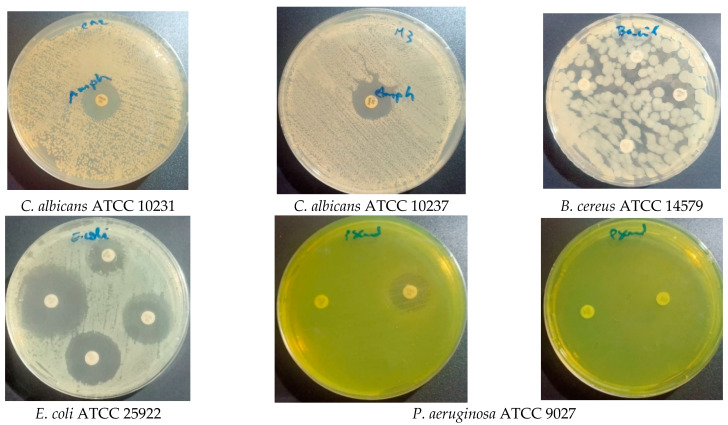

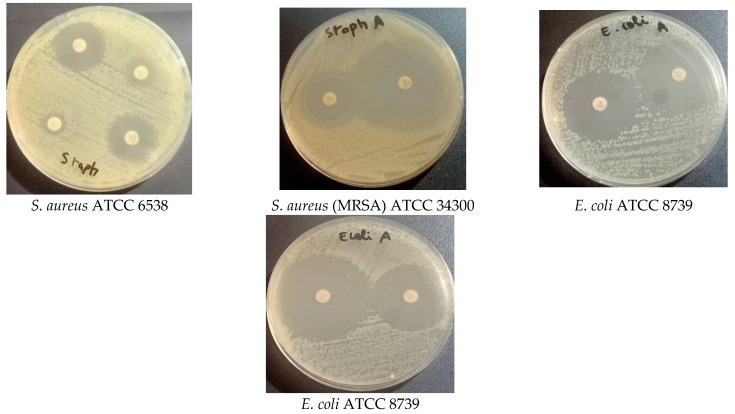
Antibacterial activity of antibiotics against the tested strains. (Amoxicillin (AMC) 30 µg, Cefazolin (CZ) 30 µg, Ceftriaxone (CRO) 30 µg, Cefoxitin (FOX) 30 µg and Amphotericin B (AMB) 20 µg).

The antimicrobial activity results of the methanolic extract of the aerial parts were obtained by the disc diffusion method, followed by measurements of the minimum inhibitory concentration (MIC), minimum bacterial concentration (MBC), and minimum fungicidal concentration (MFC) of several bacteria and yeast that are reference strains of the American Type Culture Collection (ATCC). The results are shown in Table 6 and Table 7 and Figure 4 and Figure 5 and indicate that *S. balansae* exhibited noticeable antimicrobial and antifungal activity against all tested strains with various inhibition diameters. Table 6 and Figure 4 show that all tested strains were sensitive in all the concentration ranges of 50, 100, and 200 mg/mL; the IZD was 10 to 13.33 mm for the concentration of 50 mg/mL, 11 to 15 mm for the concentration of 100 mg/mL, and 11 to 18 mm for the concentration of 200 mg/mL. *Escherichia coli*, *Staphylococcus aureus* (MRSA), and *Candida albicans* were the most sensitive for all the concentration ranges.

The MIC values were 1000 μg/mL for *Pseudomonas aeruginosa*, less than 2000 μg/mL for *S. aureus*, *Bacillus cereus*, and *C. albicans* (CA2), and 2000 μg/mL for other tested strains.

The MBC and MFC values were found to be 2000 μg/mL for all the tested strains.

The MBC/MIC ratio was 2 for *P. aeruginosa*, 1 for *S. aureus* (MRSA) and *E. coli*, and was not determined for the other strains.

The MFC/MIC ratio was 1 for *C. albicans* (M3), and it was not determined for *C. albicans* (CA2). From these results, we can conclude that the aerial parts of *S. balansae* assayed in a methanolic extract were considered bactericidal.

The antibiotic susceptibility testing results of this study (Table 6) showed that there are resistant strains such as *P. aeruginosa*, *S. aureus* (MRSA), and *B. cereus;* very sensitive strains; and extremely sensitive strains (Figure 5).

The results of this study on the in vitro antimicrobial activity of *S. balansae* as revealed in a methanolic extract, were a first. Several studies have been conducted on the antimicrobial activities of essential oils and extracts from different *Salvia* species. For instance, Tepe et al. [56] found that essential oils in *Salvia cryptantha* Montbret & Aucher ex Benth. and *Salvia multicaulis* Vahl have the capacity to inhibit the growth of pathogenic microorganisms. Another study by Tepe et al. [57] showed that a methanol extract of *S. cryptantha* possessed moderate activity against *Streptococcus pneumoniae* and *C. albicans*, whereas *S. multicaulis* had broader activity against tested microorganisms, including *S. pneumoniae*, *B. cereus*, *S. aureus*, *Moraxella catarrhalis*, *Clostridium perfringens,* and *C. albicans*. According to Longaray Delamare [58], the essential oils of *S. officinalis* L. and *Salvia triloba* L. exhibited remarkable bacteriostatic and bactericidal activities against *B. cereus*, *Bacillus megaterium*, *Bacillus subtilis*, *Aeromonas hydrophila*, *Aeromonas sobria*, and *Klebsiella oxytoca.* Also, the essential oils of both species at a concentration of 0.05 mg/100 mL methanol inhibited the growth of *S. aureus*. The growth of *S. aureus* and *A. hydrophila* was drastically reduced. The polar extracts of these species have not been studied. Ozkan et al. [59] reported the antimicrobial activity of the methanolic extract and the essential oil of *Salvia pisidica* Boiss. & Heldr. ex Benth. against 13 bacterial and 2 yeast strains. The extract (at concentrations of 5 g/100 mL or 10 g/100 mL) was effective against most of the strains tested but not against *B. cereus*, *S. aureus*, *A. hydrophila*, or the two yeast strains. The essential oils showed an antimicrobial effect against all the Gram-positive bacteria tested and against *Saccharomyces cerevisiae*, but they were not effective against all the Gram-negative bacteria and *C. albicans*. Dulger and Dulger [60] showed that the methanol extract, butanol, and chloroform fractions of *Salvia verbenaca* L. have potential antimicrobial effects against some bacteria and the yeast cultures tested, with grown inhibition area diameters in the range of 10.8 to 22.4 mm and MIC values between 0.03 and 0.34 μL/mL.

It can be concluded that this study may suggest that various extracts of Salvia species possess compounds with antimicrobial potential, which can be used as antimicrobial agents in new drugs for the treatment of infectious diseases in humans.

#### 2.6.2. Antioxidant Properties

There are several methods for determining antioxidant activity. In this study, the antioxidant properties of the methanol and petroleum ether extracts from *S. balansae* aerial parts were determined by three complementary tests: the DPPH and ABTS assays for radical-scavenging activity and the CUPRAC chelating (CUPric reducing antioxidant capacity) methods. These tests are widely used to measure the ability of natural antioxidants to transfer protons from hydrogen to free radicals. In this study, the antiradical activity was evaluated by determining the 50% inhibitory concentration (IC_50_) compared with the standard of butylated hydroxyanisole (BHA), and the results are presented in Table 8.

The antioxidant activity of *S. balansae* extracts was measured by three different methods and expressed by the IC_50_ (µg/mL) and A0.5 (µg/mL) and compared with the standard BHA. As shown in Table 8, the methanol extract was more effective than the petroleum ether extract. In fact, interesting values were noted for the DPPH, ABTS, and CUPRAC assays of 242.7 ± 7.44, 124.1 ± 9.70, and 222.9 ± 6.05 µg/mL, respectively. However, these values were still lower than the standard BHA of 1.88 ± 0.06, 3.44 ± 0.09, and 5.62 ± 0.08 μg/mL for the ABTS, DPPH, and CUPRAC assays, respectively.

Our findings exhibited a higher scavenging ability than those of Mahdjoub [27], who evaluated the hydroethanolic extract of *S. balansae* leaves and reported lower IC_50_ values of 328.95 ± 5.29 and 545.03 ± 3.267 μg/mL for the ABTS and DPPH assays, respectively. Furthermore, Mekki [26] noticed that the antioxidant values of the aqueous extract of *S. balansae* leaves, as measured by the DPPH, ABTS, and total antioxidant capacity (TAC) assays, had values higher than the standard used, which is Trolox.

It is well-known that antioxidant ability is linked to phenolic content and chemical classes. In fact, the flavonoids luteolin and catechin are widely recognized as excellent antioxidant phenolic components, comparable to the synthetic standard butylated hydroxytoluene (BHT) for neutralizing the radical DPPH [61]. In addition, ferulic acid and myricetin are well-known to scavenge free radicals by inhibiting reactive oxygen species (ROS) and chelating iron and copper ions [62]. The variations in phytochemical composition could be due to different factors, such as the climate and soil conditions, the time and period of harvesting, or the plant part used, as well as the laboratory conditions for testing, including the solvent, the temperature, and the method used for preparing the extracts. All these parameters could lead to fluctuations in the most important constituents in terms of health benefits; one must take into consideration the fact that biological activities are not only due to the main compounds of a plant extract but could also be affected by the interactions between major and minor compounds in a synergistic or antagonistic way [63].

#### 2.6.3. Enzyme Inhibition Activity

The enzyme inhibition potential of *S. balansae* against cholinesterases, α-glucosidase, and α-amylase was investigated. The plant did not exhibit enzyme inhibition activity (Table 9).

The anticholinesterase activities of the *S. balansae* methanol and petroleum ether extracts were tested using the acetyl and butyryl–cholinesterase enzymes (see Table 9). In terms of butyrylcholinesterase (BChE) inhibitory activity, the petroleum ether extract showed a significant ability, with an IC_50_ of 82.33 ± 2.13 µg/mL. Our results were in agreement with those of Rungsimakan [64], who noticed that the petroleum ether extract of *S. viridis* L. at a concentration of 10 mg/mL exhibited a more than 90% inhibitory ability against acetylcholinesterase (AChE) and BChE; however, the two extracts were less effective against AChE. Furthermore, data from the literature also revealed a low or non-existent AChE inhibitory potential of *Salvia* species. Indeed, the ethanolic extracts of *Salvia glutinosa* L., *Salvia nemorosa* L., *Salvia sclarea* L., and *Salvia pratensis* L. from Croatia did not attain a 50% efficiency [65]. Sage herbs are well-known in traditional medicine as memory enhancers, and to the best of our knowledge, there is no previous study on the cholinesterase inhibitory activity of *S. balansae* species. Recently, the evaluation of the enzyme inhibitory effects of the extracts obtained from five Turkish *Salvia* species, *S. viridis*, *Salvia wiedemannii* Boiss., *Salvia aytachii* Vural & Adıgüzel, *Salvia heldreichiana* Boiss. ex A.DC., and *Salvia aucheri* subsp. *Canescens* (Boiss. & Heldr.) Celep, Kahraman & Doğan, showed that fluctuations in the recorded values could be due to the phytochemical variability, content, and structure [66]. Cholinesterase inhibitors are the most prescribed treatment for Alzheimer’s disease, which is a permanent neurodegenerative disorder strongly linked to oxidative stress caused by the imbalance between free radicals and antioxidant content in the metabolism.

We could find no previous study on the α-glucosidase and α-amylase enzyme inhibition activities of *S. balansae*. According to our outcomes, no significant α-glucosidase and α-amylase enzyme inhibition activities (IC_50_ > 400 μg/mL) were observed in the plant extracts. It should be noted that a large number of *Salvia* species have been scarcely studied for their antidiabetic potential. A study by Nickavar and Abolhasani [67] reported that the ethanolic extract of *Salvia virgata* showed a dose-dependent α-amylase inhibition with an IC_50_ of 19.08 mg/mL. Enzyme inhibition assays performed on the *Salvia urmiensis* Bunge methanolic extract showed the highest α-glucosidase and α-amylase inhibition with the lowest IC_50_ values (IC_50_ = 8.3 and 24 µg/mL) [68]. *Salvia miltiorrhiza* Bunge is undoubtedly the most widely studied *Salvia* species in terms of its effectiveness as an antidiabetic agent [69]. The antidiabetic potential of essential oils from *Salvia* species has also been investigated in several studies, and *S. sclarea* essential oils have shown potential antidiabetic activities [70].

## 3. Materials and Methods

### 3.1. Chemicals and Spectral Measurements

Bioactivity measurements were carried out on a 96-well microplate reader, SpectraMax 340PC384, Molecular Devices (Silicon Valley, CA, USA). The measurements and calculations of the activity results were evaluated by using Softmax PRO v5.2 software. GC analyses were performed on a Shimadzu GC-17 AAF, V3, 230 V series gas chromatography (Kyoto, Japan), and GC–MS analyses were carried out on Varian Saturn 2100T (Palo Alto, CA, USA). Ethanol, petroleum ether, methanol, and boron trifluoride–methanol complex (BF3: MeOH) were obtained from E. Merck (Darmstadt, Germany). Neocuproine and ammonium acetate butylated hydroxytoluene (BHT), 1,1-diphenyl-2-picrylhydrazyl (DPPH), electrophorus electricus (Electric eel) acetylcholinesterase (AChE, Type-VI-S, EC 3.1.1.7, 425.84 U/mg), horse serum butyrylcholinesterase (BChE, EC 3.1.1.8, 11.4 U/mg), 5,50-dithiobis (2-nitrobenzoic) acid (DTNB), acetylthiocholine iodide and butyrylthiocholine chloride, galantamine were obtained from Sigma Chemical Co. (Sigma-Aldrich GmbH, Sternheim, Germany). 2,20-Azinobis (3-ethylbenzothiazoline-6-sulfonic acid) diammonium salt (ABTS) was obtained from Fluka Chemie (Fluka Chemie GmbH, Sternheim, Germany). All other chemicals and solvents were in analytical grade. For chemicals of HPLC DAD analysis, Methanol (HPLC, analytical grade) and glacial acetic acid were supplied from Merck (Sternheim, Germany). Fumaric acid (≥99.0%), gallic acid (≥99.0%), protocatechuic acid (≥99.9%), pyrocatechol (≥99.5%), catechin (≥98.0%), *p*-Hydroxybenzoic acid (≥98.0%), *p-h*ydroxybenzaldehyde (≥98.0%), 6,7-dihydroxycoumarin (≥99.0%), vanillic acid (≥97.0%), epicatechin (≥98.0%), caffeic acid (≥98.0%), chlorogenic acid (≥99.0%), *p*-coumaric acid (≥98.0%), ferulic acid (≥99.0%), cynarin (≥97.0%), coumarin (≥99.0%), prophylgallate (≥99.0%), rutin (≥94.0%), *trans*-2-hydroxycinnamic acid (≥97.0%), myricetin (≥97.0%), fisetin (≥98.0%), naringenin (≥98.0%), *trans*-cinnamic acid (≥99.0%), hesperetin (≥98.0%), genistein (≥97.0%), luteolin (≥98.0%), kaempferol (≥99.0%), apigenin (≥98.0%), *p*-hydroxy resorcinol (≥99.0%) were purchased from Sigma-Aldrich GmbH (Sternheim, Germany).

### 3.2. Plant Material

**Figure 6:**
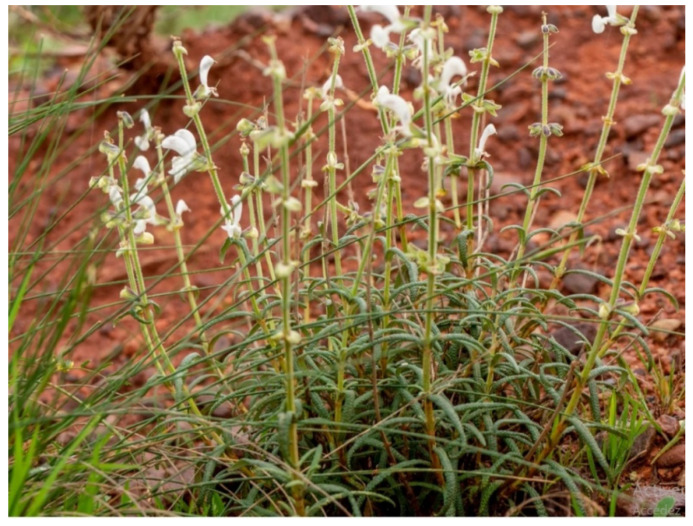
Illustration of *S. balansae* (plant habit and leaves), Mostaganem, 21 April 2022, photos Pr. Miara MD.

The fresh aerial parts samples of *S. balansae* (Figure 6) were collected during the flowering period in February 2020 from individuals spontaneously growing in the region of Mostaganem Province, Algeria, at the historical station of the plant of Algeria (40 m above sea level, GPS coordinates: N 36.030829, W 0.144134). Botanical determination was performed by Pr. MIARA Mohamed Djamel using the available literature [19]. A voucher specimen (Number N° MMD0022) has been deposited in the hernbarium of the laboratory of Botany, Ibn Khaldoun University of Tiaret-Algeria. The plant samples consisting of *S. balansae* aerial parts were washed with distilled water and then dried until a constant weight was attained. The dried samples were ground to a fine powder using an electric grinder and sieved through a 200 μm.

### 3.3. Phytochemical Screening

This is a qualitative analysis based on colorimetric reactions and/or precipitation to highlight major chemical groups. Various types of reagents have been used for this purpose. According to the appearance, the results were divided into obvious positive reaction: +++; positive reaction: ++; moderate positive reaction: +, negative reaction: −. A quantity of 5 g of plant material was macerated in 50 mL of methanol and stirred for one hour in ambient air. The mixture was filtered, and the methanolic extract was submitted to various tests. The presence or absence of different classes of secondary metabolites contained in *S. balansae* methanolic extracts were qualitatively tested as follows:

#### 3.3.1. Test for Tannins

Add a volume of 1 mL of extract to 2 mL of distilled water and 2 to 3 drops of diluted iron chloride (FeCl_3_) solution. A positive test is revealed by the appearance of a blue-black (gallic tannins), green, or blue-green color (catechic tannins) [71].

#### 3.3.2. Test for Flavonoids

Add 2.5 mL extract volume to 0.5 mL of concentrated HCl and a few small magnesium chunks (Mg). If the pink or red color develops after 3 min, that indicates the presence of flavonoids [72].

#### 3.3.3. Test for Phenolic Compounds

A volume of 10 mL of hydrogen chloride (HCl) is added to 10 mL of methanolic infusion. A positive test is revealed by the red coloration in the presence of polyphenols [73].

#### 3.3.4. Quinones

A solution of 1 mL of extract to be analyzed to which a few drops of sodium hydroxide (10% NaOH) turn yellow, indicating the presence of quinones [74].

#### 3.3.5. Anthraquinones

To 1 mL of extract to be analyzed, a few drops of potassium hydroxide (10% KOH) are introduced; after shaking, the solution turns red, indicating the presence of anthraquinones [75].

#### 3.3.6. Terpenoids

To 5 mL of extract are added 2 mL of chloroform and 3 mL of sulfuric acid (H_2_SO_4_). The formation of two phases and a brown color at interphase indicates the presence of terpenoids [74].

#### 3.3.7. Saponins

The detection of saponins is carried out by adding 1 mL of extract to 2 mL of hot water; after shaking (20 min), the appearance of a persistent foam for more than 5 min indicates the presence of saponins [71].

#### 3.3.8. Reducing Compounds

Add 1 mL of the methanolic extract to 2 mL of distilled water and 20 drops of Fehling’s liquor, then heat. A positive test is revealed by the formation of a brick-red precipitate [71].

### 3.4. Preparation of Crude Extracts

Air-dried powdered plant material (20 g) was macerated with 200 mL methanol (99.99%) (three times) and then with 200 mL petroleum ether (three times) at room temperature (25 °C) for 48 to 72 h, successively. The extracts were filtrated and evaporated under a vacuum to obtain crude extracts [75].

### 3.5. High-Performance Liquid Chromatography Analyses (HPLC-DAD)

Analysis of methanolic extract of *S. balansae* and 29 compounds of standard phenolics was performed using a high-flow liquid chromatography chain Shimadzu model (Shimadzu Cooperation, Kyoto, Japan) constituted a solvent delivery system (Shimadzu LC-20AT) and a diode array (DAD) model Shimadzu SPD-M20A and were controlled by LC-solution software v.5.96 (CBM-20A System Controller Shimadzu) (Table 10). Operating conditions were as follows: the column temperature was set to 35 °C. Chromatographic separation was performed on an Inertsil ODS-3 (4 μm, 4.0 mm × 150 mm) and an Inertsil ODS-3 guard column.

The mobile phase is composed of 0.1% acetic acid in water (A) and 0.1% acetic acid in methanol (B). Elution in mode gradient was as follows: 2% B in 3 min, 2–5% B in 3 min, 5–6% B in 2 min, 6–10% B in 4 min, 10% B in 1 min, 10–25% B in 5 min, 25–30% B in 7 min, 30–40% B in 5 min, 40–42% B in 6 min, 42–54% B in 5 min, 54–55% B in 1 min, 55–56% B in 10 min, 56–65% B in 4 min, 65–75% B in 3 min, 75–85% B in 2 min, 85–95% B in 5 min, 95% B in 2 min, 95–100% B in 1 min, 100% B in 5 min, 100–80% B in 2 min, 80–50% B in 2 min, 50–2% B in 3 min was achieved as described by Barros [76] and Tel-Çayan, [77]. The flow rate was 1.0 mL/min. The sample stock solution was prepared in methanol at a rate of 8 mg/mL. The sample and standards were filtered by an Agilent filter of 0.45 μm. Moreover, 20 μL of the sample was injected. Detection was carried out with a diode array detector (DAD) using 254 nm wavelength. Phenolic compounds detected were characterized by comparing their retention times, and the results were expressed as milligrams per gram dry weight of extract (mg/g extract).

### 3.6. HPLC-MS Analysis

The methanolic extract of *S. balansae* areal part was subjected to chromatographic analysis by high-performance liquid chromatography (HPLC) system. The HPLC YL-Clartity 9100 series is equipped with a C18 reverse-phase column consisting of silica grains grafted with a non-polar hydrocarbon unit. Reverse phase HPLC using the C18 column separated different phytochemical groups based on polarity (moderate to non-polar) in a single chromatogram. The individual phenolic compounds were quantified using a modified HPLC method according to Lee and Ong [78] and Crupi et al. [79] using gradient elution with the mobile phase consisting of 1% formic acid in water (*v*/*v*, eluent A) and 100% acetonitrile (*v*/*v*, eluent B) and was programmed in a linear gradient: A 95% (B: 5%) 0–50 min; A 5% (B 95%) after 60 min. The UV detector was set to 254 nm. Briefly, 10 mg of *S. balansae* methanol extract was dissolved in 2 mL of methanol, sonicated for 60 min, then filtered using a 0.45 µm microfilter before injecting. The sample was then injected at a flow rate of 1 mL/min, a pressure of 245 bar, and at room temperature (25 °C) into a gradient pump connected to a photodiode detector system. The separation was carried out using a C18 column (250 mm × 4.6 mm). Individual phenolic compounds were identified by comparing retention times and UV spectra of the corresponding standard compounds. Data were quantified using the corresponding calibration curves of the individual standard compound.

### 3.7. Fatty Acids Analysis by GC–MS

In a flask (25 mL), 25 mg of *S. balansea* methanolic extract was thawed in 0.5 N NaOH (2 mL). The flask was covered and warmed in a boiling water bath for five minutes, removed, allowed to cool, and 2 mL BF3-MeOH was added. The mixture was covered and heated again in a boiling water bath (80 °C) for three minutes, then left until it cooled down; 5 mL of saturated NaCl solution was added to the mixture and shaken. Finally extracted twice with *n*-hexane (20 mL) [80,81,82,83].

Qualitative and quantitative analysis of the fatty acids was performed using GC (Shimadzu GC-17 AAF, V3, 230V series gas chromatography, Japan). A Flame Ionization Detector (FID) and a DB-1 fused silica capillary non-polar column (30m × 0.25 id., film thickness 0.25 µm) were used for GC analyses of the methyl derivatives of fatty acids. Injector and detector temperatures were 250 and 270 °C, respectively. Carrier gas was Helium at a flow rate of 1.2 mL/min; sample size, 1.0 µL; split ratio, 20:1. The initial oven temperature was held at 100 °C for 5 min, then increased up to 238 °C with 3 °C/min increments and held at this temperature for 9 min. The percentage compositions of the fatty acid methyl derivatives were determined with the GC Solution computer program. The fatty acid methyl ester standard mixture (FAME Supelco™ 37 Catalog no: 47885-U) was used for the comparison and quantification of the GC chromatograms.

### 3.8. Mineral Analyses

#### 3.8.1. Sample Preparation

The sample preparation was as described by Cicero et al. [84] with some modifications; the collected samples were prepared by cleaning, slicing, and then subjecting them to a 24 h drying process at 105 degrees Celsius in an oven (Nüve, Istanbul, Turkey). After drying, the samples were homogenized using an IKA homogenizer (Staufen, Germany) and sifted through a 10-mesh sieve, resulting in an average particle size of 1600 µm. The prepared samples were stored in cleaned polyethylene bottles for subsequent analysis. Deionized water with a resistivity of 18.2 MΩ.cm^−1^, obtained from a Milli-Q^®^ system (Human Power I Plus, Sejong-si, Republic of Korea), was used for all aqueous solutions. To ensure cleanliness, all plastic and glassware were meticulously cleaned by soaking in a 10% nitric acid solution overnight and then rinsed with deionized water.

For the digestion process, a CEM Mars 5 microwave closed system (CEM, Matthews, NC, USA) was employed. The digestion of *S. balansae* involved taking 0.5 g of dry weight (dw) of the plant material, which was then ground in a Teflon mortar. This ground material was digested using a mixture of 6 mL of 65% (*v*/*v*) nitric acid (HNO_3_) and 2 mL of 30% (*v*/*v*) hydrogen peroxide (H_2_O_2_) from J.T. Baker (Mallinckrodt Baker, Milan, Italy). The mineralization process took place in an Ethos 1 digestor (Milestone, Bergamo, Italy) operating at 1000 W, with a temperature range of 150–200 °C. This temperature range was reached within 10 min and maintained for an additional 10 min. The microwave digestion followed a specific ramp (20 min), time (2 min), and power (100%) for each step. After cooling the digested samples to room temperature, they were filtered, and the resulting filtrate was diluted with 100 mL of ultra-pure water (J.T. Baker, Mallinckrodt Baker, Milan, Italy) before being stored at 4 °C. A blank digest was carried out in the same manner.

#### 3.8.2. ICP-MS Analysis

Mineral concentrations were assessed using inductively coupled plasma-optical emission spectrometry (ICP-OES) [85]. To verify the precision of the analytical method, the standard reference material (SRM) NIST-CRM-1203 Drinking Water was employed. The relative standard deviations (RSD) remained consistently below 8%. For the elemental analysis, an Agilent 7700x ICP-MS instrument was utilized. All metal concentrations were determined on a dry-weight basis. The entire process, from sample collection to analysis, was also conducted on blank samples to assess the potential for any metal contamination during the analytical procedure. The operating conditions for the ICP-MS are detailed in Table 11.

### 3.9. Biological Activities

#### 3.9.1. Antimicrobial Activities

##### Determination of Antimicrobial Activities

The antimicrobial activity of *S. balansae* methanolic extracts was determined by disc diffusion test according to a modification method described by Nicoletti et al. [86], following the Clinical and Laboratory Standards Institute (CLSI) guidelines [87], and all the equipment used was sterilized in an autoclave at 121 °C for 15 min. The test was screened against bacteria which are reference strains of the American Type Culture Collection (ATCC): Gram+ (*Staphylococcus aureus* (MRSA) ATCC 34300, *Staphylococcus aureus* ATCC 6538 and *Bacillus cereus* ATCC 14579), Gram− (*Escherichia coli* ATCC 8739, *Escherichia coli* ATCC 25922 and *Pseudomonas aeruginosa* ATCC 9027) and a yeast (*Candida albicans* ATCC 10231 (CA2) and *Candida albicans* ATCC 10237 (M3)). Bacterial strains were subjected to a continuous overnight subculture in nutrient agar and incubated at 37 °C for 24 h to optimize their growth, streaked to ensure purity in order to obtain a young culture and isolated colonies. *C. albicans* were grown on Sabouraud Dextrose Agar (SDA) for 24 h at a temperature of 37 °C. Briefly, cells were resuspended in saline (2 × 10^8^ cells/mL for bacteria (0.5 Mc Farland) and 106 cells/mL for *Candida*) and spread on the petri dishes of Mueller–Hinton Agar (MH) for bacteria and Sabouraud Dextrose Agar (SDA) for *C. albicans*. Sterile Whatman paper discs (6 mm in diameter) were placed on the surface of inoculated Petri dishes and spotted with 10 μL of 50, 100, and 200 mg/mL of methanolic extract solubilized in dimethylsulfoxide (DMSO). The Petri dishes of Mueller–Hinton Agar (MH) were incubated for 24 h at 35 ± 1 °C for bacteria and 24 h at 37 °C for C. albicans and were grown on Sabouraud Dextrose Agar (SDA). The activity was performed in triplicate, and it was determined by measuring the diameter of the growth inhibition zone (IZD) visible around the paper disc and comparing it with reference diameters related to the antibiotics used. It is limited for a diameter between 6 and 14 mm, and average for a diameter between 14 and 20 mm. For a diameter greater than or equal to 20 mm, the germ is very sensitive [88]. Negative controls were set using Wattman disks impregnated with DMSO, and the positive controls were set up with Amoxicillin (AMC) 30 µg, Cefazolin (CZ) 30 µg, Ceftriaxone (CRO) 30 µg, Cefoxitin (FOX) 30 µg, and Amphotericin B (AMB) 20 µg.

##### Determination of Minimum Inhibitory Concentration (MIC)

The MIC was evaluated by a broth microdilution method according to Okusa [89]; *S. balansae* methanolic extract was dissolved in DMSO (20 mg/250 μL) and diluted to 5 mL with Mueller–Hinton broth, the final DMSO concentration being 5%. This solution was transferred in 96-well plates (200 μL/well) and serially diluted with Mueller–Hinton broth. After an incubation period (22 h), 40 μL of a 2 mg/mL Triphenyl tetrazolium chloride (TTC) indicator solution (an indicator of microorganism growth) was added to every well, and the plate was incubated at 37 °C for about 2 h [90]. The TTC indicator solution changes from clear to purple in the presence of bacterial activity. At the same time, it remains clear when microbial growth was inhibited. The MIC was defined as the lowest concentration of methanolic extracts that showed no visible bacterial growth after incubation time (no color change (clear) of TTC).

##### Minimal Bactericidal Concentration (MBC) and Minimal Fungicidal Concentration (MFC)

The MBC and MFC were determined by directly plating the content of wells with concentrations higher than the MIC value. The MBC and MFC values were determined when there was no colony growth from the directly plated contents of the wells. The MBC and MFC were considered the lowest concentrations of methanolic extracts that killed 99.9% of microorganisms in culture on the agar plate after the incubation period. The MBC/MIC and MFC/MIC ratios were also calculated to show the nature of the antibacterial effect of methanolic extract. When the ratio was less than or equal to 4, the methanolic extract was considered bactericidal or fungicidal, and when the ratio was higher than 4, it was considered bacteriostatic or fungistatic [91].

#### 3.9.2. Antioxidant Activity

Antioxidant activities of the *S. balansae* extracts were determined by DPPH free radical scavenging, ABTS cation radical scavenging, and CUPRAC (cupric-reducing antioxidant capacity) assay spectrophotometrically using a 96-well microplate reader, SpectraMax 340PC384 (Molecular Devices, Silicon Valley, CA, USA). Softmax PRO v5.2 software (Molecular Devices, Silicon Valley) was used for calculations and measurements of the bioactivities data, and stock solutions of the samples (4000 μg/mL) were prepared.

##### Free radical Scavenging Activity DPPH Assay

The free radical-scavenging activity of the *S. balansae* extracts was determined by the DPPH assay [92]. Briefly, 39.4 mg of DPPH was dissolved in methanol to make a 0.1 mM solution. Moreover, 40 μL of each extract at different concentrations was added to 160 μL DPPH (0.1 mM in methanol). The resulting solution was vigorously agitated, and its absorbance was measured at 517nm after one hour of incubation in the dark at room temperature. BHA was used as a standard antioxidant for comparison of the activity. For each sample, the measurements were performed in triplicate. Ability to scavenge the DPPH was as follows: Scavenging activity % = [Abs (control) − Abs (sample)]/Abs (control) × 100. Abs control is the initial concentration of the DPPH, and Abs sample is the absorbance of the remaining concentration of DPPH in the presence of the extract and positive control. The findings were given as half-maximal inhibitory concentration (IC_50_) (g/mL), which reflected the extract concentration necessary to inhibit DPPH by 50%.

##### ABTS Cation Radical Decolorization Assay

The ABTS scavenging activity was measured with the [93] technique. The ABTS was created by reacting 7 mM ABTS in water with 2.45 mM potassium persulfate and storing it in the dark at room temperature for 12 h. Before use, the ABTS solution was diluted with distilled water to achieve an absorbance of 0.70 (±0.01) at 734 nm using a spectrophotometer (Shimadzu 1601, Japan). In each well of the microplate, 160 μL of this solution is added to 40 μL of the sample at different concentrations. Methanol served as a negative control, and BHA was used as a standard antioxidant. The absorbance was read at 734 nm after 10 min incubation at room temperature. For each sample, the measurements were carried out in triplicate. The results were expressed as IC_50_. The sample concentration that provided a 50% ABTS scavenging effect (IC_50_) was estimated using a graph showing the ABTS scavenging effect percentage vs. sample concentration.

ABTS scavenging effect % = ((A Control − A Sample)/A Control) × 100

where A Control is the starting ABTS concentration, and A Sample is the absorbance of the residual ABTS concentration in the presence of the extract and positive control.

##### Cupric Reducing Antioxidant Capacity (CUPRAC)

The CUPRAC activity of *S. balansae* extracts was determined spectrophotometrically using a 96-well microplate reader according to the method of Apak et al. [94] modified by Öztürk et al. [95]. This method is based on the reduction of Cu^2+^ followed by the treatment of Cu^+^ with a chromogenic reagent Neocuproine (NC) (2,9-dimethyl-1,10-phenanthroline) in the presence of phenolic hydroxyls leading to the formation of a stable complex between neocuproine and copper ions (Cu^+^) that absorbs at 450 nm. A mixture consisting of 60 μL of NH_4_Ac ammonium acetate (1 M, pH = 7.0), 50 μL of 7.5 mM neocuproine, and 50 μL of copper chloride (CuCl_2_·2H_2_O) 10 mM was prepared, followed by 40 μL of sample solution at different concentrations was added to the initial mixture. After one hour of incubation at room temperature, the absorbance is read at 450 nm. BHA was the standard antioxidant used in this assay. For each sample, the measurements were carried out in triplicate. The results are expressed as absorbances, and A0.5 values (μg/mL) corresponding to the concentration indicating an absorbance intensity of 0.50 are calculated and compared to those of BHA used as positive control.

#### 3.9.3. Enzyme inhibition Activity

##### Anticholinesterase Activity

In vitro spectrophotometric methods were used to determine acetylcholinesterase (AChE) and butyrylcholinesterase inhibitory (BChE) [96,97] activities of *S. balansae* extracts by using a 96-well microplate reader, SpectraMax 340PC384 (Molecular Devices, Silicon Valley, CA, USA). Softmax PRO v5.2 software (Molecular Devices, Silicon Valley) was used for calculations and measurements of the bioactivities data. Stock solutions of the samples at 2000 μg/mL concentration were prepared. The Ellman test is a standard protocol for the determination of free thiols; it is based on the cleavage of acetylthiocholine by AChE and butyrylthiocholine by BChE to generate thiocholine, which reacts with 5,5′-dithiobisnitrobenzoate (DTNB) to form the 5-thio-2-nitrobenzoate anion of yellow color. In the presence of an enzymatic inhibitor, this reaction allows us to study the kinetic parameters and, thus, determine the IC_50_ values (concentration decreasing 50% of the enzymatic activity). In this method, the key substrates for the reaction were iodide acetylthiocholine iodide and butyrylthiocholine chloride, and electric eel AChE and horse serum BChE were used. Briefly, 20 μL of AChE enzyme (5.32 × 10^−3^ U) or BChE (6.85 × 10^−3^ U) were incubated for 15 min at 25 °C with 150 μL of buffer with 0.1 M sodium phosphate (pH 8.0) and 10 μL of sample at different concentrations. The reaction was then started by adding 10 μL of iodide acetylthiocholine (7.1 × 10^−4^ M) or butyrylthiocholine chloride (2 × 10^−4^ M) and 10 μL of DTNB (5 × 10^−4^ M). The absorbance of the yellow 5-thio-2-nitrobenzoate anion produced is measured at 412 nm every five minutes for 15 min. The positive control used was galanthamine at the same concentrations as the samples. The percentage inhibition of AChE and BChE is determined relative to the blank (methanol with phosphate buffer pH = 8) by the formula:

Inhibition = [(Enzyme activity without extract − Enzyme activity with the extract)/Enzyme activity without extract] × 100

##### α-Amylase/α-Glucosidase Inhibitory Activities

The α-amylase/α-glucosidase inhibitory activities of *S. balansae* extracts were determined spectrophotometrically [98,99] by using a 96-well microplate reader, SpectraMax 340PC384 (Molecular Devices, Silicon Valley, CA, USA). Softmax PRO v5.2 software (Molecular Devices, Silicon Valley) was used for calculations and measurements of the bioactivity data.

1. Determination of α-amylase inhibitory activity

α-Amylase inhibitory activity of the extracts was tested by using the method previously reported by Quan et al. [99] with slight modifications in the use of incubation time, reagents, and amounts of the used reagents and samples. Moreover, 25 µL sample solution and 50 µL α-amylase solution (0.1 units/mL) in phosphate buffer (20 mM pH = 6.9 phosphate buffer prepared with 6 mM NaCl) were mixed in a 96-well microplate. The mixture was pre-incubated for 10 min at 37 °C. After pre-incubation, 50 µL starch solution (0.05%) was added and incubated for 10 min at 37 °C. The reaction was completed by the addition of 25 µL HCl (0.1 M) and 100 µL Lugol solutions. Moreover, 96-well microplate reader was used to measure absorbance at 565 nm. Acarbose was used as standard. The sample concentration providing 50% inhibition activity (IC_50_) was calculated from the graph of α-amylase inhibitory activity against sample concentrations.

2 Determination of α-glucosidase inhibitory activity

α-Glucosidase inhibitory activity of the extracts was determined using the methodpreviously reported by Kim et al. [98] with slight modifications in the use of incubationtime, reagents, and amounts of the used reagents and samples. A total of 50 µL phosphate buffer (0.01 M pH = 6.9), 25 µL PNPG (4-N-nitrophenyl-a-D-glucopyranoside) in phosphate buffer (0.01 MpH = 6.9), 10 µL sample solution, and 25 µL α-glucosidase (0.1 units/mL) in phosphate buffer (0.01 M pH = 6.0) were mixed in a 96-well microplate. The mixture was incubated for 20 min at 37 °C. A total of 90 µL sodium carbonate (0.1 M) was added to the microplate to end the reaction. A 96-well microplate reader was used to measure absorbance at 400 nm. Acarbose was used as standard. The sample concentration providing 50% inhibition activity (IC_50_) was calculated from the graph of α-glucosidase inhibitory activity against sample concentrations.

### 3.10. Statistical Examinations

The results of all biological activities were the average of three separate studies. The data were reported as mean standard error, which means (±SEM). Student’s *t*-test was used to evaluate significant differences between means; *p*-values of 0.05 were considered significant.

## 4. Conclusions

The incredible diversity of bioactive phytochemicals found in medicinally valuable plants is a precious treasure conferred by nature. In our study, we delved into the phytochemical composition, antioxidant potential, enzyme inhibition properties, and antimicrobial effects of both methanolic and petroleum ether extracts obtained from the aerial parts of *S. balansae*. Our results revealed the richness of phytochemical constituents in the aerial parts of *S. balansae*, which also exhibited a high mineral content. An HPLC-DAD analysis indicated the presence of 12 distinct phenolic compounds, while an HPLC-MS examination quantified 9 phenolic constituents. The petroleum ether extract displayed a fatty acid profile consisting of 17 compounds, with palmitic acid as the predominant fatty acid. Comparing the two extracts, the methanol extract exhibited more potent antioxidant activity. These findings underscore the fact that the *S. balansae* plant is a bountiful source of bioactive compounds. These results are both encouraging and inspiring, prompting the need for further research to isolate and purify individual components responsible for these activities and to explore the potential for in vivo biological effects.

## Figures and Tables

**Table 1:** Phytochemical screening results of *S. balansae* methanolic extract.

	Plant	Results
Compounds		
Phenolic compounds		+
Flavonoids		+
Tannins (Gallic)		+
Quinones		++
Anthraquinones		++
Terpenoids		+
Saponins		+++
Reducing compounds		+++

(+++): strongly positive reaction, (++): positive reaction, (+): weakly positive reaction.

**Table 2:** Mineral contents of the aerial parts of *S. balansae* and NIST-CRM 1203 Drinking Water (mg/Kg).

	Plants	*S. balansae*	Certified and Experimental Values of Studied Metals in NIST-CRM 1203 Drinking Water (mg/kg). ^a^		
Mineral Contents					
			Certified Value (mg/kg)	Experimental Value ± S.D. (mg/kg) ^b^	Recovery Value (%)
Phosphorus (%)		0.24 ± 0.00 *	-	-	-
Potassium (%)		2.38 ± 0.07 *	-	-	-
Calcium (%)		1.52 ± 0.15	99.78 ± 0.50	100.42 ± 0.95	100.64
Magnesium (%)		0.45 ± 0.03	99.77 ± 0.50	100.68 ± 1.02	100.23
Iron (mg L^−1^)		1160.36 ± 23.20 *	200.3 ± 1.0	199.89 ± 2.05	99.94
Copper (mg L^−1^)		19.53 ± 0.97	2000 ± 10	202.9 ± 0.12	101.45
Manganese (mg L^−1^)		66.22 ± 2.64 *	50.17 ± 0.25	50.02 ± 0.75	99.67
Zinc (mg L^−1^)		67.43 ± 2.02 *	1000 ± 5	1003.1 ± 7.8	102.59
Boron (mg L^−1^)		30.16 ± 0.60 *	-	-	-

* Values expressed herein are mean ± SEM of three parallel measurements *p* < 0.05. ^a^ Ten times dilution of Certified NIST-CRM 1203 Drinking Water. ^b^ Average of triplicate measurements of certified material (*p* < 0.05).

**Table 3:** Phenolic composition of the aerial part methanolic extract of *S. balansae* by HPLC-DAD analysis.

Peak N°	Compounds	RT (min)	DAD Spectra Values	μg/g Extract
**1**	Protocatechuic acid	24.625	259, 294	50
**2**	*p*-Hydroxybenzoic acid	30.867	208, 255	60
**3**	6,7-Dihydroxycoumarin	33.435	208, 257, 298, 345	tr
**4**	Vanillic acid	34.758	208, 260, 292	180
**5**	Caffeic acid	35.280	215, 324	tr
**6**	*p*-Coumaric acid	40.874	208, 309	tr
**7**	Ferulic acid	42.564	216, 233, 324	450
**8**	Coumarin	45.178	278, 309	tr
**9**	*trans*-2-Hydroxycinnamic acid	48.243	212, 276, 325	tr
**10**	*trans*-Cinnamic acid	56.203	204, 275	tr
**11**	Luteolin	57.872	254, 350	480
**12**	Kaempferol	62.485	255, 363	130

tr: trace amount detected.

**Table 4:** HPLC-MS analysis of the phenolic composition of the methanolic extracts of *S. balansae*.

N	RT (min)	Area (mV.s)	Height (mV)	Area (%)	Height (%)	W05 (min)	Compound Name
**1**	3.460	85.937	7.920	0.7	5.1	0.14	Sinapinic acid
**2**	3.603	10.603	1.968	0.1	1.3	0.09	Nicotinamide
**3**	4.613	9.040	0.603	0.1	0.4	0.12	Ascorbic acid
**4**	16.590	2686.466	15.792	21.7	10.1	3.24	Myricetin
**5**	19.060	131.010	5.637	1.1	3.6	0.48	Butylated hydroxyanisole
**6**	20.403	156.513	7.668	1.3	4.9	0.25	Epicatechin
**7**	21.667	111.398	3.643	0.9	2.3	0.52	genistein
**8**	23.097	85.366	4.143	0.7	2.7	0.27	Kaempferol
**9**	28.863	8965.095	101.406	72.5	65.1	0.63	Catechin
**10**	31.190	124.939	6.918	1.0	4.4	0.24	ND
	Total	12366.366	155.697	100.0	100.0		

ND: not determined.

**Table 5:** Fatty acid composition determined by GC/MS in *S. balansae* petroleum ether extract.

Peak N°	RT	RI ^a^	RI ^b^	Compound Name	Concentration (%)	Literature
**1**	16.723	1431	1452.5	Dimethyl phthalate	4.80	Lopez-Avila et al. [44]
**2**	19.026	1443	1473	Buthylatedhydroxytoluen	0.55	Turchini et al. [45]
**3**	19.69	1499	1486	Dihydroactinidiolide	1.51	Senatore et al. [46]
**4**	20.489	1540	1540.9	Nonanedioic acid (Azaleic acid)	0.77	Sanches-Silvia et al. [47]
**5**	25.262	1689	1689	Isobutyl methyl phthalate	3.11	Liang [48]
**6**	27.251	1725	1723	Myristic acid (C14:0)	2.24	Rout et al. [49]
**7**	31.568	1842	1847	Phytone	1.92	Kowalski [50]
**8**	32.429	1863	1864	Diisobutyl phthalate	1.33	Lopez-Avila et al. [44]
**9**	34.418	1938	1926	Palmitic acid (C16:0)	36.72	Robinson et al. [51]
**10**	35.143	1943	1949	Isophytol	0.86	Rout et al. [52]
**11**	36.815	1995	1996	15-Methyl-hexadecanoic acid	0.49	da Camara et al. [53]
**12**	39.923	2090	2096	Lineloic acid (C18:2)	9.39	Rout et al. [52]
**13**	40.126	2095	2106	Oleic acid (C18:1)	12.62	Rout et al. [49]
**14**	40.963	2128	2128	Stearic acid (C18:0)	8.97	Rout et al. [49]
**15**	46.993	2324	2321.8	Eicosanoic acid (C20:0)	8.37	Remberger et al. [54]
**16**	52.657	2531	2531	Docosanoic acid (C22:0)	0.78	Kowalski [50]
**17**	58.747	2600	2600	*n*-Hexacosane	0.97	Fuentes [55]

^a^ Kovats index on DB–5 fused silica column. ^b^ Kovats index on DB–5 fused silica column in literature.

**Table 6:** Antibacterial activity of *S. balansae* aerial part methanolic extract.

		Strains		*S. aureus* (MRSA) ATCC 34300	*S. aureus* ATCC 6538	*P. aeruginosa* ATCC 9027	*E. coli* ATCC 8739	*E. coli* ATCC 25922	*B. cereus* ATCC 14579	*C. albicans* (CA2) ATCC 10231	*C. albicans* (M3) ATCC 10237
Extract											
***S. balansae***	Diameter of inhibition zone (mm) *		50 (mg/mL)	13 ± 0.66	10 ± 1.11	10 ± 0	13.33 ± 0.44	10.66 ± 0.44	10.66 ± 0.44	10.66 ± 0.44	12.33 ± 0.44
			100 (mg/mL)	14 ± 0	11 ± 0	13 ± 0	14 ± 0	11 ± 0	11 ± 0	12 ± 0	15 ± 0
			200 (mg/mL)	16 ± 0	11 ± 0	14 ± 0	17 ± 0	12 ± 0	11 ± 0	15 ± 0	18 ± 0
	MIC (μg/mL)			2000	<2000	1000	2000	2000	<2000	<2000	2000
	MBC (μg/mL)			2000	2000	2000	2000	2000	2000	-	-
	MFC (μg/mL)			-	-	-	-	-	-	2000	2000
	MBC/MIC ratio			1	Nd	2	1	1	Nd	-	-
	MFC/MIC ratio			-	-	-	-	-	-	Nd	1

* Values expressed herein are mean ± SEM of three parallel measurements. Nd: not determined.

**Table 7:** Antibacterial activity of antibiotics against the tested strains.

	Strains	*S. aureus* (MRSA) ATCC 34300	*S. aureus* ATCC 6538	*P. aeruginosa* ATCC 9027	*E. coli* ATCC 8739	*E. coli* ATCC 25922	*B. cereus* ATCC 14579	*C. albicans* (CA2) ATCC 10231	*C. albicans* (M3) ATCC 10237
Antibiotics									
inhibition zone (mm)	Amoxicillin (AMC) 30 µg	40	22	00	34	20	15	-	-
	Cefazolin (CZ) 30 µg	00	23	00	34	25	00	-	-
	Ceftriaxon (CRO) 30 µg	00	15	18	44	36	00	-	-
	Cefoxitin (FOX) 30 µg	22	12	00	40	25	00	-	-
	Amph (AMB) 20 µg	-	-	-	-	-	-	22	20

**Table 8:** Antioxidant activities (IC_50_ and A0.5 values) of *S. balansae* methanol and petroleum ether extracts.

Extracts and Compounds	Antioxidant Activity		
	DPPH Assay ^a^	ABTS Assay ^a^	CUPRAC Assay ^a^
	IC_50_ (µg/mL)	IC_50_ (µg/mL)	A0.5(µg/mL)
Methanol extract	242.7 ± 7.44	124.1 ± 9.70	222.9 ± 6.05
Petroleum ether extract	NA	NT	NT
BHA ^b^	3.44 ± 0.09	1.88 ± 0.06	5.62 ± 0.08

Abbreviation: BHA, 2-tert-Butyl-4-hydroxyanisole and 3-tert-butyl-4-hydroxyanisole. ^a^ Values expressed herein are mean ± SEM of three parallel measurements. *p* < 0.05. ^b^ Reference compound. NA: not active. NT: not tested.

**Table 9:** Anticholinesterase and antidiabetic activities (IC_50_ values) of *S. balansae* methanolic petroleum ether extracts.

Extracts	Anticholinesterase Activity		Antidiabetic Activity	
	AChE Assay ^a^	BChE Assay ^a^	α-Amylase Inhibitory Assay ^a^	α-Glucosidase Inhibitory Assay ^a^
	IC_50_ (µg/mL)	IC_50_ (µg/mL)	IC_50_ (µg/mL)	IC_50_ (µg/mL)
Methanol extract	>200	>200	>400	>400
Petroleum ether extract	>200	82.33 ± 2.13	>400	>400
Galantamine ^b^	4.31 ± 0.03	45.29 ± 0.06	-	-
Acarbose ^b^	-	-	Nd	Nd

^a^ Values expressed herein are mean ± SEM of three parallel measurements. *p* < 0.05. ^b^ Reference compound. Nd: not determined.

**Table 10:** Retention time, calibration curves, regression coefficient (R2), linearity ranges, LODs, and recoveries of phenolic standards at 254 nm.

No	Compounds	RT ^a^ (min)	Calibration Equation	R2 ^b^	Linear Range (μg/mL)	λmax, nm	LOD ^c^ (μg/mL)	LOQ ^c^ (μg/mL)	Recovery (%)	RSD ^d^ within Day (*n* = 7)	RSD between Days (*n* = 7)
**1**	Protocatechuic acid	24.625	y = 76181x − 88801	0.9995	3.13–100	254	3.42	10.35	102.35 ± 4.21	3.19	1.22
**2**	4-Hydroxybenzoic acid	30.867	y = 111102x + 21691	0.9993	1.56–50.0	254	1.58	4.79	100.82 ± 3.89	4.00	2.41
**3**	6,7-Dihydroxycoumarin	33.435	y = 34377x − 32740	0.9940	5.00–50	254	3.98	12.07	104.11 ± 5.06	4.94	3.72
**4**	Vanillic acid	34.758	y = 74653x − 9634.1	0.9998	1.56–100	254	1.56	4.68	103.58 ± 4.43	5.06	3.88
**5**	Caffeic acid	35.280	y = 67972x − 32965	0.9880	3.00–30.0	254	4.54	13.75	102.67 ± 4.92	4.01	5.87
**6**	*p*-Coumaric acid	40.874	y = 18300x + 6153.3	0.9998	6.25–400	254	5.46	16.56	101.60 ± 2.36	3.14	0.44
**7**	Ferulic acid	42.564	y = 35737x + 12977	0.9999	2.34–300	254	3.96	11.99	100.99 ± 3.54	3.20	0.51
**8**	Coumarin	45.178	y = 36021x − 23215	0.9999	3.13–100	254	2.21	6.69	101.74 ± 4.83	3.59	1.08
**9**	*trans*-2-Hydroxycinnamic acid	48.243	y = 53843x + 124308	0.9996	3.13–400	254	3.09	9.27	99.75 ± 3.75	2.85	0.75
**10**	*trans*-Cinnamic acid	56.203	y = 87505 + 4540.2	0.9999	1.25–50.0	254	0.58	1.74	100.85 ± 1.58	5.78	5.66
**11**	Luteolin	57.872	y = 9895.8x + 159212	0.9950	4.84–620	254	2.75	8.34	100.00 ± 4.91	2.88	2.39
**12**	Kaempferol	62.485	y = 68024x + 7902.3	0.9999	1.56–100	254	1.01	3.06	98.57 ± 3.84	1.87	5.04

^a^ RT: Retention time of the compound in minutes, ^b^ R2: linearity of the calibration graph, ^c^ LOD: Limit of Detection in μg/mL and LOQ: Limit of Quantification in μg/mL, ^d^ RSD: Percentage relative standard deviation.

**Table 11:** ICP-MS instrumental operating conditions.

Instrument	Agilent™ 7700x ICP-MS
RF power	1600 W
RF match	2.10 V
Sampling depth	10.0 nm
Nebulizer gas	0.57 L/min
S/C temperature	2 °C
Nebulizer type	MicroMist
Spray chamber	Scott-type double-pass
Ar flow rate	Plasma: 15 L/min; Auxiliary: 0.9 L/min; Nebulizer: 1.0–1.1 L/min
Solution uptake rate	1.8 mL/min
VacuumInterface	4 torr, quadrupole: 2 105 torr
Data acquisition	Peak hopping; Replicate time 200 ms; Dwell time 200 ms; Sweeps/reading 3; Readings/replicate 3; Number of replicates 3

## Data Availability

All the data in the article are available from the corresponding author upon reasonable request.
